# Estimating and reporting treatment effects in clinical trials for weight management: using estimands to interpret effects of intercurrent events and missing data

**DOI:** 10.1038/s41366-020-00733-x

**Published:** 2021-01-18

**Authors:** Sean Wharton, Arne Astrup, Lars Endahl, Michael E. J. Lean, Altynai Satylganova, Dorthe Skovgaard, Thomas A. Wadden, John P. H. Wilding

**Affiliations:** 1 Weight Management and Diabetes, The Wharton Medical Clinic, Hamilton, ON Canada; 2 Department of Health and Kinesiology, York University, Toronto, ON Canada; 3 Department of Nutrition, Exercise and Sports, University of Copenhagen, Copenhagen, Denmark; 4 Novo Nordisk A/S, Søborg, Denmark; 5 School of Medicine, Dentistry and Nursing, University of Glasgow, Royal Infirmary, Glasgow, UK; 6 Department of Psychiatry, Perelman School of Medicine, University of Pennsylvania, Philadelphia, PA USA; 7 Department of Cardiovascular and Metabolic Medicine, Institute of Life Course and Medical Sciences, University of Liverpool, Liverpool, UK

**Keywords:** Randomized controlled trials, Weight management

## Abstract

In the approval process for new weight management therapies, regulators typically require estimates of effect size. Usually, as with other drug evaluations, the placebo-adjusted treatment effect (i.e., the difference between weight losses with pharmacotherapy and placebo, when given as an adjunct to lifestyle intervention) is provided from data in randomized clinical trials (RCTs). At first glance, this may seem appropriate and straightforward. However, weight loss is not a simple direct drug effect, but is also mediated by other factors such as changes in diet and physical activity. Interpreting observed differences between treatment arms in weight management RCTs can be challenging; intercurrent events that occur after treatment initiation may affect the interpretation of results at the end of treatment. Utilizing estimands helps to address these uncertainties and improve transparency in clinical trial reporting by better matching the treatment-effect estimates to the scientific and/or clinical questions of interest. Estimands aim to provide an indication of trial outcomes that might be expected in the same patients under different conditions. This article reviews how intercurrent events during weight management trials can influence placebo-adjusted treatment effects, depending on how they are accounted for and how missing data are handled. The most appropriate method for statistical analysis is also discussed, including assessment of the last observation carried forward approach, and more recent methods, such as multiple imputation and mixed models for repeated measures. The use of each of these approaches, and that of estimands, is discussed in the context of the SCALE phase 3a and 3b RCTs evaluating the effect of liraglutide 3.0 mg for the treatment of obesity.

## Introduction

Research programs investigating weight-loss interventions are currently based almost exclusively on randomized clinical trials (RCTs) to ascertain the efficacy and safety of a treatment. Randomization provides the highest level of evidence for these evaluations, by ensuring that neither the intervention being investigated nor the comparator by default is favored through potential effect modifiers at the time of randomization. However, effect modifiers or confounding factors may emerge after randomization that affect the estimated differences between the treatment groups. Hence, while the integrity of the randomization must be respected in any analysis of an RCT, the question on what to do about post-randomization effect modifiers remains and must be discussed.

Regulators require estimates of the treatment effect to be reported, usually provided as the placebo-adjusted mean (the difference in total weight loss between treatment arms) [1, 2]. At first glance, this may seem an appropriate and straightforward way to quantify the treatment effect, but there are several reasons why the placebo-adjusted effect sizes of a drug in RCTs do not necessarily indicate the effect sizes that are achieved in real-life patient management. For example, pharmacotherapy for obesity produces weight loss not only by a simple drug action, but by increasing the extent to which individuals can make changes in their diets and physical activities. Importantly, participants in weight management RCTs are seeking the effect of the treatment, and can themselves see their progress with weight change, which can alter their adherence and other behaviors. In addition, there are many external factors that can influence weight change, so observed differences in RCTs should be interpreted in the light of various factors or events that can occur during a trial. For example, individuals receiving placebo may not experience satisfactory weight loss and as a result may adopt another behavioral measure or weight-loss medication (other than the active medication) during the trial, and thus experience a larger reduction in body weight than if they had only received placebo. Events that occur during a trial that may influence interpretation of the results are termed *intercurrent events* [3].

In addition to intercurrent events, missing body weight assessments at the end of treatment need to be handled in the statistical analysis. The various approaches to handling missing data each have their own advantages and disadvantages, and will in some cases rely on unverifiable assumptions [4]. Analytical methods required by regulatory bodies, such as the US Food and Drug Administration (FDA) and the European Medicines Agency (EMA), may be appropriate from a regulatory perspective, but may not provide the most useful information to inform clinicians about the treatment effects that could be expected in their patients. Furthermore, data presented in prescribing information and scientific publications for the same product may have been analyzed using different statistical approaches and thereby yield different efficacy results, making it challenging to communicate the treatment effect to the relevant audiences.

There is a need for greater transparency and alignment in how weight management RCTs are designed, conducted, and reported to help different stakeholders (e.g., clinicians, regulatory bodies, prescribers, patients, payers, and guideline developers) understand how estimated treatment effects should be interpreted. One way to address this need is to utilize estimands. The estimand concept [3] provides a framework to align trial design and objectives to ensure that the estimated treatment effect is relevant to the scientific question(s) of interest. The attributes that determine how the treatment effect must be interpreted, and thus define an estimand, are: (i) the treatment condition of interest; (ii) the population to be studied; (iii) the endpoint of interest (including whether a participant experiences an intercurrent event); and (iv) the population-level summary (i.e., how the treatment effect is to be determined). Estimands aim to provide information for the target population on what the efficacy outcomes might be expected to be in the same patients under different conditions [3]. In addition to stating that estimands should be used, the International Council for Harmonisation of Technical Requirements for Pharmaceuticals for Human Use (ICH) guidelines also emphasize the importance of considering intercurrent events when defining the scientific question of interest and underline key principles for handling missing data [3].

In line with the EMA and FDA regulatory guidelines [5, 6], ongoing and newly initiated weight management trials will be required to report results using estimands. The SCALE (Satiety and Clinical Adiposity-Liraglutide Evidence in individuals with and without diabetes) phase 3b trials investigating efficacy and safety of liraglutide 3.0 mg as an adjunct to diet and lifestyle advice for weight management have recently been published with two estimands [7, 8]. Similarly, clinical trial programs for weight management drugs in development, such as the semaglutide 2.4 mg once-weekly STEP (Semaglutide Treatment Effect in People with obesity) trial program [9–16] are planned to report results utilizing estimands.

In this review, we discuss how intercurrent events can affect the estimation and interpretation/quantification of a treatment effect in weight management trials; how approaches for handling missing data have evolved over the last decade; and implications for interpreting clinical data from weight management RCTs. We examine these issues using the SCALE phase 3a clinical trial program as an example and address how the use of estimands may help provide greater transparency in clinical trial reporting.

## Intercurrent events in weight management trials and their impact on data analysis and interpretation

**Fig. 1:**
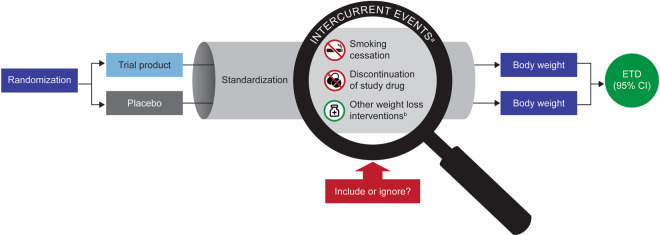
Intercurrent events and their implications in weight management clinical trials. *CI* confidence interval, *ETD* estimated treatment difference. ^a^Intercurrent events are likely to be unbalanced between treatment arms, potentially introducing bias. ^b^Adopting other weight-loss medication recorded as concomitant medication during the trial. Non-pharmacological measures could also be considered, e.g., actively engaging in additional weight-loss programs such as joining a gym or commercial weight-loss program, or bariatric surgery.

RCTs are the gold standard for demonstrating a treatment’s efficacy and safety, where many factors are standardized through inclusion criteria and guidance on what to do during the trial (i.e., the trial protocol). Randomization of trial participants into treatment groups aims to avoid selection bias and to balance the treatment groups at baseline. However, after randomization, clinical trial participants can experience intercurrent events that affect the endpoint of interest and its interpretation, and they may be unequal between randomized treatment groups (Fig. 1). It is therefore important to understand intercurrent events that are common during weight management trials and how they may influence the analysis and interpretation of results.

It is vital to recognize that in weight management RCTs, the treatment effect (weight loss) is desired by the trial participants, and that it is not only a direct pharmacological effect of trial product but is also mediated by changes in eating and/or activity behaviors. Trial participants can see, and feel, the effect of a treatment and can become aware of their allocation to active or placebo arms. Participants allocated to placebo groups are increasingly likely to withdraw from treatment or to adopt additional measures aimed at weight loss. During a trial, participants may thus discontinue their randomized treatment for various reasons, such as lack of tolerability, disappointment in the weight loss achieved, relocation, health complications, or initiation of other medication, etc. In addition, some participants might be switched to a different weight-loss medication (or even undergo bariatric surgery) if they do not achieve weight reductions as sought for medical or personal reasons, or as expected from the protocol [17]. Smoking aggravates most medical complications of obesity, and cessation is often concurrently recommended; however, smoking cessation usually leads to weight gain, often substantial [18, 19], and could therefore differentially affect body weight if participants decided to stop smoking during the trial [19–21]. Smoking cessation may be more successful with an active weight-loss medication than with a placebo, thus reducing the apparent effect size of an active medication. Such intercurrent events will likely result in different placebo-adjusted treatment effects than if they had not occurred and raise questions about how these events should be accounted for and how trial results should be interpreted (Table 1).

**Table 1:** Intercurrent events in weight management clinical trials, their potential impact on body weight, and interpretation of results.

When estimating the placebo-adjusted weight loss in a clinical trial, investigators need to decide whether weight measurements taken after intercurrent events should be included or excluded from a particular analysis. For example, including weight measurements in the analysis for those who discontinued treatment reflects how, in a real-world situation, adherence to any treatment is likely to vary between patients. If post-discontinuation weight measurements were excluded from a particular analysis and replaced by model predictions, the treatment effect would represent a hypothetical situation in which all participants tolerated and adhered to the treatment. Such hypothetical estimation may not represent a real-world situation but provides information, and a different perspective, on the extent of weight loss that can be achieved in the target population if the drug is taken as intended and no intercurrent events occur.

### Which intercurrent events should be accounted for in weight management RCTs?

Intercurrent events are not limited to the examples discussed so far, and it may not always be clear what should be categorized as an intercurrent event. For instance, trial participants might seek other weight-loss interventions such as joining a gym or a commercial weight-loss program. Investigators would need to decide whether engaging in these activities would be part of the diet and exercise regimen detailed in the trial protocol or if such activities should be regarded as intercurrent events that need to be addressed in the statistical analysis. These alternative weight-loss interventions may not be equally available or affordable to people with obesity in routine clinical practice, but might be adopted more by trial participants if they are made available as part of the trial. When designing a trial, consideration must be given to scenarios that could be identified as intercurrent events and which of these events would be relevant to that particular trial. Discontinuation of randomized medication is common and may substantially affect weight loss; consequently, treatment discontinuation should always be handled as an intercurrent event. In contrast, intercurrent events such as smoking cessation or switching to another approved weight-loss medication would only need to be accounted for in trials in which these phenomena are expected to have a substantial effect on the endpoint in question, for example, if the intercurrent event is common, or if it occurs at different rates between active and placebo arms. Crucially, intercurrent events should be prespecified and the strategy for analyzing the data in the presence of these events should be relevant to the underlying clinical question of the trial.

## Missing data in weight management trials

In weight management RCTs, not all participants remain in the trial for the planned duration (e.g., due to lack of tolerability, dissatisfaction with treatment, or being lost to follow up). In one weight management observational study comparing characteristics between completers and non-completers, dropout rates were reported to be 21% at 1 month and 57% at 6 months [22]. These losses will result in missing data if post-dropout measurements are not obtained, which can reduce statistical power and create challenges in analyzing and interpreting an incomplete dataset [23].

The problem of missing data is best handled by minimizing the number of subjects lost to follow up through various means such as trial design (e.g., continuing to collect data for participants even if they discontinue their randomized treatment), and providing guidance on statistical approaches to handling missing data [24, 25]. For example, to help ensure that participants attended scheduled visits, trial protocols for SCALE phase 3a trials of liraglutide 3.0 mg for weight loss included reminders at each visit to book the next appointment [26, 27]. In the recent SCALE Intensive Behavioral Therapy phase 3b trial, participants were encouraged to attend scheduled visits and thus have their weight measurements recorded regardless of discontinuing treatment [8]. To promote participant retention, the SCALE Insulin phase 3b trial permitted individuals to stop and restart liraglutide 3.0 mg without re-escalating the dose, or with re-escalation if three consecutive doses had been missed [7].

Useful information can be applied from the known effect of treatment discontinuation at the end of the trial in other participants. In some cases, it is possible to obtain follow-up data from other sources, such as routine medical records, which can inform projections for those who discontinue early or who are lost to follow up [17, 28]. It might be expected that participants who drop out of clinical trials of weight management would not go on to achieve further weight loss, but some might be successful with their weight loss outside of the trial setting [28].

## Estimating treatment effects: accounting for intercurrent events and handling missing data

The ICH recommends the use of estimands in clinical trials [3]. Utilizing estimands in the context of weight management RCTs ensures that anticipated intercurrent events of relevance, such as smoking cessation, adding or switching to another approved weight-loss medication, or discontinuation, are prespecified.

Different scientific questions may be addressed, and therefore multiple estimands employed, in a single clinical trial. The *treatment policy* estimand strategy, outlined in the ICH guidelines [3], provides a population-level effect of the tested therapy, which may be of primary interest to policy-makers. In contrast, the hypothetical strategy, outlined in the ICH guidelines [3] (also known as the *trial product* estimand in some clinical trials [29]), provides information on the pharmacological effect of the tested therapy under the assumption that intercurrent events will not occur. It is important to ensure that an appropriate description of the estimands used is provided when weight management RCT results are communicated. This will help the audience gain a greater understanding of how the results have been estimated and how they should be interpreted, and aids comparison of results with those from other clinical trials. In addition to estimands, guidelines state that the most appropriate statistical methods should be selected to account for intercurrent events [3] and missing data [24], so that the treatment effect of interest is the one that is eventually estimated.

Primary outcomes for RCTs in weight management include mean weight loss from randomization to end of treatment and placebo-adjusted weight loss. Mean values for these outcomes can be calculated from observed data or from imputed data when there are missing observations. A completers analysis uses data from a subset of participants who did not have their endpoint imputed in the primary analysis and can bypass the issue of missing data. Different methods of statistical analysis can be employed to estimate the treatment effect of interest, taking into account intercurrent events and missing data; all could be considered valid under appropriate circumstances but for different specific purposes. Such analytical methods include the last observation carried forward (LOCF), multiple imputation (MI), and mixed model for repeated measures (MMRMs) [23]. Statistical tests for whether differences in the following treatment effect estimates are significant are not easy to interpret since the estimates have different interpretations and provide different clinical perspectives on the treatment effect. Thus, such tests should be avoided.

### Treatment effects based on data obtained from treatment completers

A frequently used method to estimate the effect in randomized trials is through restricting the analysis set to comprising only data from patients who continue the randomized treatment until the scheduled end-of-treatment visit (i.e., treatment completers). This approach simplifies the statistical analysis and circumvents the problem of missing data, and is often thought to provide an estimate of the pharmacodynamic effect of the intervention under investigation (i.e., the effect of the intervention when used as intended). However, the underlying causes for discontinuing randomized treatment often depend on the treatment itself. For instance in the SCALE trials, non-completing patients typically discontinued liraglutide 3.0 mg due to tolerability issues, whereas non-completing patients typically discontinued placebo due to lack of effect, and there are no strong arguments to suggest that the patients who discontinued placebo would also have been the patients who would experience tolerability issues had they been randomized to liraglutide 3.0 mg instead. Hence completer analyses are subject to different selection mechanisms across the randomized arms, and consequently violate the integrity of the randomization. Thus, the completer analysis should in general be avoided in the reporting of results from randomized trials.

### Treatment effects estimated by LOCF-based analyses

Historically, participants in weight management trials who discontinued their allocated treatment were often censored from the trial, resulting in no or few post-discontinuation weight assessments available for the statistical analysis [24]. This means that the *treatment policy* estimate of the placebo-adjusted weight loss could not be obtained without making extensive and unverifiable assumptions in the statistical analysis. In some studies, in which treatment discontinuation resulted in missing data, post-discontinuation assessments were imputed using the LOCF approach [26, 27, 30–32].

The LOCF approach uses the participant’s last observed value (e.g., body weight at last visit before discontinuation) for the endpoint analysis. The last observed value can either replace a missing weight assessment for a trial participant lost to follow up or replace weight assessments that were taken after discontinuation of the active treatment or placebo, if the participant continued to have weight assessments taken at pre-determined times while off treatment. LOCF-based placebo-adjusted weight-loss estimations can consequently be interpreted in one of two ways depending on whether LOCF values are used only to impute missing data, or if they are used to impute missing data and replace off-treatment values. When imputing missing data only, the underlying assumption is that body weight would be unaffected by discontinuation for trial participants lost to follow up. Participants typically experience weight regain after discontinuing an effective treatment [33]; therefore, the assumption of no weight change post-discontinuation required by this version of the LOCF approach is unrealistic.

The imputation of missing values and replacing off-treatment values with the last observation on-treatment carried forward was the approach used in the SCALE phase 3a weight management publications [26, 27, 32]. In this approach, the last observation on-treatment carried forward-based treatment effect can be interpreted as the effect of the active treatment as long as participants adhered to treatment. The clinical relevance of the treatment effect estimated using last observation on-treatment carried forward will be limited if trial participants tend to discontinue placebo earlier (or later) than participants in the active treatment group [4] since there is a risk of not comparing like with like in this type of analysis. LOCF is of some statistical value to determine efficacy, but it is of limited value if a real-life treatment-effect size is required.

### Treatment effects estimated using MI

With the increasing computational awareness and capacity to develop statistical methods that provide more useful interpretations of the treatment effect, MI methods have become dominant for the reporting of recent weight management clinical trials (e.g., semaglutide obesity phase 2 trial [34] and the SCALE phase 3b trials [7, 8]). MI is a computationally intensive method that can replace missing weight measurements for participants lost to follow up with the most plausible value that would have been expected if the participants had been available for the end-of-trial weight assessment. Reflecting the uncertainty around what those values might have been, the MI procedure introduces random “noise” to the imputed values and generates numerous new datasets (sometimes hundreds, or even thousands); hence, the term “multiple” imputation. The reported placebo-adjusted weight loss is the average of all these different datasets where, for each dataset, it is estimated with a corresponding 95% confidence interval. The number of datasets generated should be enough to ensure that if the MI was to be repeated with a new random noise, it would provide approximately the same result. The reported placebo-adjusted weight loss therefore depends on the collected data and the assumption around the expected body weight for participants lost to follow up, but does not depend on the random noise introduced in the imputation procedure.

A typical assumption for the expected body weight for participants lost to follow up would be that these participants, on average, weigh the same at the end of the trial as comparable participants (by age, sex, occupation, family structure, etc.) who discontinued treatment at a similar time, but remained in the trial and thus had a final weight measurement. This kind of MI is sometimes called “*sampling from retrieved dropouts*” [4]. Another assumption that is sometimes used is that participants in the active arm lost to follow up would weigh the same, on average, as comparable participants randomized to placebo. This kind of MI, a more conservative one, is sometimes called “jump to reference” [35]. Further approaches toward imputing more realistic results might include gathering body weight data on dropouts from other datasets [28] or potentially by using data from similar participants in similar trials of the same product. Regardless of the assumption used to impute missing values, the MI approach is considered valid to evaluate a placebo-adjusted weight loss for a *treatment policy estimate* because all available data are used irrespective of what happens to the participants in the trial (i.e., ignoring intercurrent events).

The supplementation of the available data with imputed values for participants lost to follow up ensures that all randomized participants contribute to the effect estimate and thereby preserves the integrity of the randomization. However, the imputation in itself does not affect the interpretation of the placebo-adjusted weight loss.

### Treatment effects estimated using MMRM

To supplement the *treatment policy* estimand, weight management clinical trials may report the placebo-adjusted weight loss that is expected when adhering to the treatment at the investigated dose under the assumption of no intercurrent events [3]. To obtain an estimate of the placebo-adjusted weight loss if all participants had adhered to the treatment for the duration of the trial, all weight assessments after treatment discontinuation are excluded from the analysis. A frequently used statistical technique to obtain this estimate with a corresponding 95% confidence interval is the MMRM.

The MMRM technique borrows information from participants who are still adhering to the randomized medication to obtain a plausible estimate of what the placebo-adjusted weight loss would have looked like in the hypothetical situation in which all participants were assumed to adhere to the assigned treatment to the same degree as those who were reported to remain adherent throughout the trial. Thus, the MMRM analysis method estimates the hypothetical treatment effect under average or normal adherence.

While the assumption of no intercurrent events is often not entirely realistic, this estimate is the closest we can currently get to an estimate of the true pharmacological effect of the drug and is referred to as the *trial product estimate*.

### Examples of different imputation methods used in the SCALE phase 3a clinical trial program in weight management

The different analysis methods discussed here, such as completers, LOCF, MMRM, and MI, may produce differing results in a given clinical trial. Here, we present the example of the SCALE phase 3a program, investigating the efficacy and safety of liraglutide 3.0 mg for weight management, to demonstrate this, as different methods of analysis were required by the EMA compared with the FDA (Table 2). At the time of the respective regulatory submissions, the EMA required an LOCF-based approach [36] whereas the FDA requested an MI approach [37].

**Table 2:** Difference in treatment effect across the SCALE phase 3a trials estimated by LOCF, MI, and MMRM^a^.

	Estimated treatment difference (placebo-adjusted weight loss) from baseline, % (95% CI)			
	Completers [38]	LOCF-based (EU SmPC) [36]	MI (US PI) [37]	MMRM [38]
SCALE obesity and prediabetes [27]	−5.7 (−6.3, −5.1)	−5.4 (5.8, −5.0)	−4.5 (−5.2, −3.8)	−5.8 (−6.3, −5.3)
SCALE diabetes [26]	−4.1 (−5.3, −2.9)	−4.0 (−4.8, −3.1)	−3.7 (−4.7, 2.7)	−4.4 (−5.5, −3.3)^b^
SCALE maintenance [32]	NA	−6.1 (−7.5, −4.6)	−5.2 (−6.8, −3.5)	−6.1 (−7.7, −4.6)

*CI* confidence interval, *EMA* European Medicines Agency, *EU* European Union, *FDA* Food and Drug Administration, *LOCF* last observation carried forward, *MI* multiple imputation, *MMRM* mixed model for repeated measures, *NA* not available, *SCALE* Satiety and Clinical Adiposity-Liraglutide Evidence in individuals with and without diabetes, *US* United States.

^a^The estimation of LOCF and MMRM is based on all data until last observation on treatment and extrapolating the remaining data until the planned end of treatment at week 56. Across the three trials data was extrapolated for ~31% of patients. The amount of extrapolation for each patient was determined by the time of treatment discontinuation. For the MI-based analysis, only weight assessments missing at week 56 were imputed and across the trials ~22% of patients had a missing weight assessment at week 56.

^b^MMRM analysis results for liraglutide 3.0 mg.

The SCALE phase 3a trials discussed in this paper were designed prior to the recent regulatory requirements for intercurrent events and hence patients discontinuing randomized injections or violating the protocol were withdrawn from the trials and only invited in for a landmark visit to assess the body weight. Hence the estimations of LOCF and MMRM are based on all data until last observation on treatment and extrapolating the remaining data until the planned end of treatment at week 56 (Table 2).

Across three phase 3a SCALE trials [26, 27, 32], the treatment effects estimated using an LOCF-based approach were larger than those estimated using an MI approach (Table 2). This is because MI-based results reflect the treatment effect of liraglutide 3.0 mg after 56 weeks in a population in which some participants discontinued treatment and thus regained body weight, which often occurs after discontinuation of a weight-loss medication [32, 33]. Therefore, the MI-based analysis takes into account the effects of treatment discontinuation.

For the purpose of this review, separate MMRM analyses were performed for the three phase 3a SCALE trials [38] and, in general, the treatment effects estimated using the MMRM analysis method were larger than those estimated using LOCF (Table 2). These larger estimates with the MMRM analysis method would be expected since MMRM-based estimates in the SCALE phase 3a trials reflect the treatment effect of liraglutide 3.0 mg after 56 weeks in a hypothetical situation in which all randomized patients were adherent for the planned duration of the trial. In contrast, LOCF-based estimates in these trials reflect the treatment effect for liraglutide 3.0 mg up to the time point of discontinuation, which could be less than 56 weeks. In addition, treatment effects estimated using MMRM analyses are typically larger than those seen with MI analyses since the MMRM estimates the treatment effect while participants are adhering to the investigational drug, whereas MI-based estimates (reflecting the intent-to-treat approach) are irrespective of adherence.

Results were similar when sensitivity analyses were conducted according to completers, MMRM, and MI (Table 2), indicating the plausibility of the imputation methods. However, since the completers analysis compromizes the integrity of randomization, interpretation should be made with caution.

### Interpreting trial results in real-world clinical practice

Treatment effects reported in prescribing information may differ for the same product depending on the country/region and the preference of different regulatory agencies for reporting different analyses. Moreover, analyses and treatment effects in prescribing information may also differ from those reported in publications in medical journals, where the choice of analyses may be dictated by authors and/or journal editors and reviewers. Therefore, clinicians should be aware of the analyses used when interpreting and trying to compare data from prescribing information and/or publications.

Real-world evidence can supplement the interpretation of the placebo-adjusted treatment effect by comparing the weight loss achieved in the RCT in question with what has been obtained with the investigational drug in a clinical database or register. For example, a recent real-world evidence study assessed weight loss in patients receiving liraglutide 3.0 mg in combination with diet and exercise. In this study, overall mean weight loss in patients known to be persistent on liraglutide 3.0 mg was reported to be −6.3% at 4 months after initiation, −7.1% at 6 months after initiation, and −6.5% at 6 months for all subjects regardless of persistence [39]. In the phase 3a SCALE trials, participants’ body weight loss with liraglutide 3.0 mg treatment was 6.0–8.0% reported using a LOCF-based analysis method [26, 27, 32] and 4.9–7.4% using MI [37]. Although it is not possible to draw direct comparisons from the real-world evidence study and the RCTs, these examples demonstrate how reporting the treatment effect from different perspectives can provide a broader understanding of the weight loss that can be expected with a medication, and can be useful for clinicians when assessing pharmacotherapeutic options for their patients. It is possible that in the future, sources of real-world evidence data in combination with RCTs will provide the best estimates of real-life treatment effects.

## Reporting results from weight management clinical trials: moving forward

To avoid selection bias, either all randomized participants must contribute to the placebo-adjusted weight loss or comparable groups must be selected in the two treatment groups in the estimation. LOCF-based statistical approaches are no longer recommended due to concerns regarding the plausibility of the assumptions and the potential for bias [24]. However, the introduction of the estimand concept has shifted the discussion from potential bias to whether the LOCF-based estimate of the treatment effect has a clinically relevant interpretation.

Clinicians and their patients may want to know the potential effect on weight loss when fully adhering to the treatment, i.e., the extent of the pharmacological effect. Likewise, it is useful to estimate weight loss in a population that might not have been fully adherent due to various reasons, including adverse events and treatment discontinuation, i.e., an average population effect. To provide different perspectives on the estimated treatment effect in weight management RCTs, we recommend that future publications investigating new or already-approved weight-loss medications report the *treatment policy estimand* and the *trial product estimand*. We also recommend that the estimand concept should be applied to new trial designs to ensure transparency and facilitate interpretation of data and comparison across trials. While estimands do not solve all problems of trial design and interpretation, this new concept will enable trials to be designed more appropriately to obtain relevant information to address the clinical question(s) of interest. The SCALE phase 3b clinical trials have been reported [7, 8], building on the existing evidence for the efficacy and safety of liraglutide 3.0 mg in weight management, and are some of the first clinical trials in weight management to report results using estimands.

## Other considerations in weight management clinical trial reporting

The use of the estimands concept in weight management trials opens up wider discussions about estimating and communicating treatment effects for weight management interventions. The placebo-adjusted weight loss is required by regulatory bodies to determine whether an investigational drug is efficacious and tolerable but does not provide sufficient information on the effect size in clinical practice, since it can never fully account for real-life variability. Therefore, the placebo-adjusted weight loss does not necessarily provide the most useful information to translate into clinical practice. Similarly, clinicians, policy-makers, and people with obesity may find the mean weight loss of a treatment of little practical or planning value, while the proportions of people achieving categorical weight loss of ≥5, ≥10, and ≥15% might be more relevant to inform real-world clinical practice. Categorical weight-loss outcomes can also be combined with treatment adherence in order to estimate the composite effect of treatment adherence and weight loss. While the aim of this article is not to review these and other considerations in detail, the box below represents a compilation of a few unanswered questions that could guide further academic and clinical discourse in this area.

Ongoing questions for weight management clinical trial reporting

- In clinical practice, what is the best way for clinicians to interpret and communicate results from weight-loss trials?
- Is the proportion of people achieving categorical weight losses more useful for healthcare payers, guideline developers, clinicians, and patients than the placebo-adjusted mean?
- Should trial protocols have clearer definitions of what are classed as protocol violations, (e.g., initiation of another weight-loss intervention) and how they should be handled in analyses?

## Summary

In weight management RCTs, the trial outcome is desired and visible to participants and treatment blinding may not be fully achievable with modern potent adjunctive pharmacotherapy. Intercurrent events such as smoking cessation, switching to or adding another weight-loss treatment, or treatment discontinuation, can be unbalanced between treatment arms and impact the endpoint of interest, and subsequent data analysis and interpretation. Analyses should adopt appropriate statistical methods to take into account intercurrent events and missing data. LOCF-based methods are no longer considered appropriate for weight management clinical trials and are being replaced by MI- and MMRM-based analysis methods. Regulatory requirements for the conduct, analysis, and reporting of weight management trials have evolved in recent years, indicating the need to provide different perspectives on the treatment effect when reporting trial results. The use of estimands in weight management clinical trials is recommended to allow for easier understanding and interpretation of clinical trial results among stakeholders. Two estimands will be more prevalent in future publications for weight management RCTs: the *treatment policy estimand* (estimated using MI) and the *trial product estimand* (estimated using MMRM).

## Supplementary information

Supplementary Appendix
