# Supplementary material for: Estimating and reporting treatment effects in clinical trials for weight management: using estimands to interpret effects of intercurrent events and missing data
Source: Int J Obes (Lond). 2021 Jan 18;45(5):923–33. doi: 10.1038/s41366-020-00733-x (PMC8081661; doi:10.1038/s41366-020-00733-x)
Supplement: Supplementary file 1 — Supplementary Appendix [file 41366_2020_733_MOESM1_ESM.pdf]

Supplementary information

Appendix. US Food and Drug Administration. Statistical assessment of efficacy. Background information for advisory committee on liraglutide for weight management. Statistical briefing material for the Endocrinologic and Metabolic Drugs Advisory Committee, September 11, 2014.

U.S. Department of Health and Human Services
Food and Drug Administration
Center for Drug Evaluation and Research
Office of Translational Sciences
Office of Biostatistics

STATISTICAL ASSESSMENT OF EFFICACY

BACKGROUND INFORMATION FOR ADVISORY COMMITTEE ON LIRAGLUTIDE FOR WEIGHT MANAGEMENT

Statistical briefing material for the Endocrinologic and Metabolic Drugs Advisory Committee, September 11, 2014

Bradley W. McEvoy, DrPH
Mark Rothmann, PhD

Division of Biometrics II
Office of Biostatistics
Office of Translational Sciences
Center for Drug Evaluation and Research
U.S. Food and Drug Administration

Document Date: August 13, 2014

1 INTRODUCTION AND BACKGROUND

Novo Nordisk (the sponsor) has submitted a new drug application (NDA) for liraglutide 3.0 mg/day as an adjunct to a reduced caloric diet and physical exercise for chronic weight management in adult patients that are overweight with co-morbidities or obese. This document summarizes the primary efficacy findings from five randomized Phase 2 and Phase 3 Trials included in the NDA. This review mainly focuses on the three Phase 3 trials (1839, 1922 and 1923) due to their weight management objective, trial duration (at least 56 weeks), and their ability to support our preferred analysis. For these trials an emphasis is placed on 1) the extent and impact of missing data, and 2) the statistical methods used to explore the potential impact of missing data.

This document is organized as follows.

- Section 2 discusses statistical considerations of two elements of the 2007 Draft FDA Guidance for weight management—efficacy benchmarks and analysis methods.
- Section 3 summarizes the individual trial designs, statistical methods, patient disposition and trial results. In Section 3.3 limitations of the sponsor's missing data sensitivity analyses are explored and discussed. In Section 3.4 the primary prespecified analysis is shown to over-estimate the intention-to-treat (ITT) effect using our preferred analysis by a relative change of up to 15%. Our preferred approach is an ITT analysis that represents missing data on the primary endpoint using information from subjects that prematurely discontinued but returned for a primary endpoint measurement. Based on this approach (detailed in Section 3.3), subjects treated with liraglutide 3.0 mg compared to placebo, had an average excess reduction from baseline to week 56 in fasting weight of 4.8% (95% CI =4.3, 5.3) in Trial 1839 and 3.4% (95% CI =2.3, 4.5) in Trial 1922. When liraglutide was used after an initial 5% weight reduction from a low caloric diet in Trial 1923, liraglutide treated subjects lost on average an additional 5.3% (95% CI =3.8, 6.8) compared to placebo.
- Section 4 provides a brief summary of findings.

The statistical evaluation of cardiovascular events is addressed in a separate statistical review conducted by the Division of Biometrics VII.

2 Draft FDA Guidance for products for weight management: Statistical considerations

In 2007 FDA released the Draft Guidance for Industry: *Developing Products for Weight Management* that provides recommendations for the development of drugs for the indication of weight management. The content relevant to evaluating the effectiveness of liraglutide is described in the sections on efficacy benchmarks and statistical methods. Below excerpts from these sections are provided along with a discussion of statistical considerations.

***Efficacy benchmarks*:**

Box-1. Efficacy Benchmarks (Section IV.B.3.c)
In general, a product can be considered effective for weight management if after 1 year of treatment either of the following occurs:<br>• The difference in mean weight loss between the active-product and placebo-treated groups is at least 5 percent and the difference is statistically significant.<br>• The proportion of subjects who lose greater than or equal to 5 percent of baseline body weight in the active-product group is at least 35 percent, is approximately double the proportion in the placebo-treated group, and the difference between groups is statistically significant.

It is useful to consider the benchmarks within the context of the goal of a product for weight management: long-term reduction in fat mass with a goal of reducing morbidity and mortality. It must therefore be recognized that the effectiveness is evaluated using a surrogate endpoint. Whether the observed change in the surrogate is clinically meaningful depends, in part, on safety considerations. That is, whether the benefits outweigh the risks relies on weighting the demonstrated effectiveness of the product against its risks.

***Analysis methods*:**

Box-2. Analysis Methods (Section VI.C)
The analysis of (percentage) weight change from baseline should use ANOVA or ANCOVA with baseline weight as a covariate in the model. The analysis should be applied to the last observation carried forward on treatment in the modified ITT population defined as subjects who received at least one dose of study drug and have at least one post-baseline assessment of body weight. Sensitivity analyses employing other imputation strategies should assess the effect of dropouts on the results. The imputation strategy should always be prespecified and should consider the expected dropout patterns and the time-course of weight changes in the treatment groups. No imputation strategy will work for all situations, particularly when the dropout rate is high, so a primary study objective should be to keep missing values to a minimum. Repeated measures analyses can be used to analyze longitudinal weight measurements but should estimate the treatment effect at the final time point.

Since the publication of the Draft Guidance the Division's view and handling of missing data has evolved, which was communicated to the sponsor in a May 06, 2013 Advice letter. The letter stated while the Division was not requesting the primary analysis be modified, the Division has reconsidered the use of LOCF following the publication in 2010 of a report on missing data by the National Academy of Sciences (NAS), The "*Prevention and Treatment of Missing Data in Clinical Trials.*"

An analysis that uses the last available observation on-treatment (LAO-OT) presents unique challenges interpreting the results overall and relative to the estimate of the intention-to-treat (ITT) effect. Some of the challenges associated with the recommended analysis are:

- Part of a therapy's effect is mitigated through the ability to tolerate the therapy. Therefore, an analysis that excludes observations after discontinuing therapy likely inflates the treatment effect since subjects that go off-treatment tend to regain weight.
- The average endpoint may have limited utility for a patient making a treatment decision because it is not known (nor is it possible to know) how long they will tolerate treatment; this can only be known after starting a treatment.
- The endpoint may not be clinically relevant for subjects with limited treatment adherence (e.g., one or two months) given the long-term goals of weight management.
- The distribution of the timing of the last available on-treatment measurement can differ across treatment arms. When this occurs the comparison of on-treatment experiences across treatment arms can be time-confounded.

Based on these considerations our preferred analysis is one that estimates the intent-to-treat effect using data from all subjects at the landmark visit. Because none of the sponsor's sensitivity analyses were found to adequately estimate this quantity for reasons described in Section 3.3, we fit two different statistical models to estimate this quantity; details of these model are provided in Section 3.3.

3 Evaluation of Efficacy

3.1 Study Design and Endpoints

A summary of the study design and endpoints for the trials reviewed in this document are shown in Table 1. Additional details of the trial designs are provided in Sections 3.1.1 to 3.1.5 with primary efficacy endpoints described in Section 3.1.6. Across the Phase 3 trials the studies differed in important ways. In particular, Trial 1922 was the only study in subjects with type 2 diabetes mellitus (T2DM); Trial 1923 studied subjects after having lost 5% of their bodyweight during a 12 week low calorie diet (LCD); and Trial 3970 primary objective was not related to inducing or maintaining weight loss. In all trials subjects received diet and activity counseling.

Table 1. Summary of Trial Designs

Trial	Study population	Design	Length of study (primary landmark visit)	Primary endpoints	Treatment arm (No. randomized)
1807 (Phase 2)	Obese subjects w/o T2DM	R, DB/OL*, PG, AC, PC	104 weeks (week 20)	1. Δ in bodyweight (kg)<br>2. 5% responder	Lira 1.2 mg –95<br>Lira 1.8 mg –90<br>Lira 2.4 mg –93<br>Lira 3.0 mg –93<br>Placebo – 98<br>Orlistat –95
1839 (Phase 3)	Non-diabetic subjects that are obese or overweight with co-morbidities	R, DB, PG, PC	160 weeks (week 56)	1. Δ in bodyweight (%)<br>2. 5% responder<br>3. 10% responder	Lira 3.0 mg –2487<br>Placebo –1244
1922 (Phase 3)	Obese or overweight subjects with T2DM	R, DB, PG, PC	56 weeks (week 56)	1. Δ in bodyweight (%)<br>2. 5% responder<br>3. 10% responder	Lira 1.8 mg –211<br>Lira 3.0 mg –423<br>Placebo –212
1923 (Phase 3)	Obese subjects without diabetes	R, DB, PG, PC	56 weeks (week 56)	1. Δ in bodyweight (%)<br>2. maintain run-in bodyweight<br>3. 5% responder	Lira 3.0 mg –212<br>Placebo –210
3970 (Phase 3)	Non-diabetic, obese subjects with moderate or severe sleep apnea	R, DB, PG, PC	32 weeks (week 32)	1. Δ in AHI	Lira 3.0 mg –180<br>Placebo –176

Source: FDA statistical reviewer
T2DM-Type 2 diabetes mellitus; R-Randomized; DB-Double-blind; PG-Parallel group; PC-placebo controlled; AC-active controlled; OL-open-label.
* DB/OL: the active control arm was open-label, and the liraglutide and placebo arms were double-blind.

3.1.1 Trial 1807

Trial 1807 was a Phase 2, randomized, partially blinded, parallel group, placebo and active controlled dose-finding trial in non-diabetic, obese subjects. A total of 564 subjects in 19 sites in 8 European countries were randomized 1:1:1:1:1:1 to one of four liraglutide doses (1.2, 1.8, 2.4, or 3.0 mg once daily), matching liraglutide placebo, or open-label orlistat (120 mg three times daily). Randomization was stratified by gender. The treatment duration was planned for 20 weeks with an optional 84 week extension period. A total of 398 randomized subjects consented to and continued study treatment in the extension phase. After the 52 week visit subjects treated with liraglutide or placebo were initially treated with the open-label 2.4 mg dose. Subjects were subsequently switched to the 3.0 mg dose following discussion from the planned week 52 analysis.

3.1.2 Trial 1839

Trial 1839 was a randomized, double-blind, placebo controlled, parallel group trial in non-diabetic obese or overweight subjects with co-morbidities. A total of 3731 subjects in 191 sites including 69 in the US were randomized 2:1 to liraglutide 3.0 mg or placebo. Randomization was stratified by pre-diabetes status (with, or without) and BMI (≥ 30 kg/m^2, or < 30 kg/m^2). Subjects in the pre-diabetes stratum were randomized to 160 weeks of treatment; data post 56

weeks was not included in the submission. Subjects in the stratum without pre-diabetes were randomized to 56 weeks of treatment followed by a 12 week re-randomization treatment period. Subjects randomized to liraglutide were then re-randomized 1:1 to liraglutide or placebo. Subjects that prematurely discontinued were asked to attend a follow-up visit that took place 56 weeks after their randomization date.

3.1.3 Trial 1922

Trial 1922 was a 56 week randomized, double-blind, placebo controlled, three-arm parallel group trial in obese or overweight subjects with T2DM. A total of 846 subjects in 126 sites including 67 in the US were randomized 2:1:1 to liraglutide 3.0 mg, liraglutide 1.8 mg or placebo as an add-on to their background diabetes treatment. Randomization was stratified by HbA1c (≥ 8.5%, or < 8.5%) and background treatment (diet and exercise or single compound oral antidiabetic treatment, or combination oral antidiabetic treatment). Subjects that prematurely discontinued were asked to attend a follow-up visit that took place 56 weeks after their randomization date.

3.1.4 Trial 1923

Trial 1923 was a 56 week randomized, double-blind, placebo controlled parallel group trial in non-diabetic obese or overweight subjects with dyslipidemia and/or hypertension. Subjects were randomized if they lost at least 5% of their bodyweight during a 12 week low calorie diet (1200–1400 kcal/day) run-in period. A total of 422 subjects in 36 sites in the US (26) and Canada (10) were randomized 1:1 to liraglutide 3.0 mg or placebo. Randomization was stratified by co-morbidity status (presence or absence of treated or untreated hypertension or dyslipidemia). Subjects that prematurely discontinued were asked to attend a follow-up visit that took place 56 weeks after their randomization date.

3.1.5 Trial 3970

Trial 3970 was a 32 week randomized, double-blind, placebo controlled parallel group trial in non-diabetic obese subjects with moderate or severe obstructive sleep apnea (OSA). The primary study objective was to evaluate whether liraglutide reduces the severity of OSA assessed by apnea-hypopnoea index (AHI). A total of 359 subjects in 40 sites in the US (35) and Canada (5) were randomized 1:1 to liraglutide 3.0 mg or placebo.

3.1.6 Efficacy Endpoints

The pre-specified primary efficacy endpoints for the individual trials are displayed in the table below. Note that for Trial 1839 the fourth primary endpoint is still being collected at the time of the NDA submission; interim results are not presented in this review. Furthermore, it is noted that the primary endpoint definition from trial protocols (fixed time-point) is not consistent with the endpoint in the primary analysis that relies on LAO-OT. This lack of harmonization not only can lead to results being misinterpreted, it is also problematic for this submission because the treatment effect estimated from the primary analysis is found to over-state the estimated ITT treatment effect using our preferred approach.

The primary efficacy endpoints of percent change in fasting body weight from baseline and 5% responders is consistent with what is described in the Draft FDA Guidance. The 10% responder

endpoint (Trials 1839 and 1922) is not described in the Guidance but is included due to different regulatory requirements for the European Medicines Agency.

In Trial 3970 AHI is captured during an overnight visit using polysomnography. An AHI event is characterized by either a transient reduction in, or cessation of breathing. The criteria for an event are included in the Appendix. Importantly, the ability to establish benefit by comparing the average change in AHI rate between treatment groups is limited because, as noted by the sponsor (protocol, page 82) "clinical relevant change in AHI has not been established."

Table 2. Primary efficacy endpoints by trial

Trial ID	1st primary	2nd primary	3rd primary	4th primary
1839, 1922 (at week 56)	Change in fasting body weight from baseline (%)	Proportion of subjects losing at least 5% of fasting baseline body weight (5% responders)	Proportion of subjects losing at least 10% of fasting baseline body weight (10% responders)	Onset of type 2 diabetes in subjects with pre-diabetes (at week 160)
1923 (at week 56)	Change in fasting body weight from baseline (%)	Proportion of subjects that maintained the ≥ 5% reduction in initial fasting body weight achieved during the low calorie diet run-in period	Proportion of subjects losing at least 5% of fasting baseline body weight (5% responders)	-
1807 (at week 20)	Change in fasting body weight from baseline (kg)	Proportion of subjects losing at least 5% of fasting baseline body weight (5% responders)	-	-
3970 (at week 32)	Change in AHI rate (events per hour)	-	-	-

Source: FDA statistical reviewer

3.2 Patient Disposition and Missing Data

Patient Disposition: Patient disposition is summarized for the individual trials in Table 12 in the Appendix. A large proportion of subjects, 29%, withdrew from the Phase 3 trials prior to study specific landmark visit. This observation is not unexpected for weight management programs. In the placebo group the proportion of discontinuations was greater overall than in the liraglutide arms. Across the Phase 3 trials the key reasons for study discontinuation were as follows:

- *Adverse Events*: Adverse events accounted for 9.5% of early study discontinuations in the liraglutide arms compared to 4.1% in the placebo arms. In the liraglutide arm discontinuation tended to occur shortly after randomization.
- *Withdrawal Criteria:* In Trials 1839, 1922 and 1923 study discontinuations due to withdrawal criteria are non-specific and comprise several components including consent withdrawal, pregnancy, and target dose not tolerated. The majority of study discontinuations criteria were consent withdrawal. Subjects in the placebo group were more likely have a withdrawal related to withdrawal criteria than liraglutide.
- *Ineffective Therapy*: A small number of overall discontinuations were attributed to Ineffective Therapy (liraglutide 3.0 mg, 25 subjects; placebo, 42 subjects). From a sampling of subjects in Trial 1839 that discontinued for reasons other than this, several

commented on the ineffectiveness of the therapy (Table 13 in the Appendix). The extent to which this occurred in Trial 1839 and the other trials is not known.

In Trial 1807, 472 or 84% of the 564 randomized subjects completed the 20 week main treatment period, with 74 of them not enrolling into the 84 week extension period. The decision not to continue follow-up appears to be associated with degree of weight loss at week 20, with the subjects that enrolled in the extension having more favorable average weight reductions than those that did not (Table 3). This trend was consistent across study arms except for the 1.2 mg liraglutide dose.

Table 3. Mean change from baseline (kg) at week 20 by missing status and enrollment into the 84 week extension period (Trial 1807).

**Consented for 84 week extension**	**Yes**		**No**		**No**	
**Weight at week 20**	**Available**		**Available**		**Missing**	
**Treatment Group**	**N**	**Mean Change**	**N**	**Mean Change**	**N**	**Mean Change***
Liraglutide 1.2 mg	68	-5.5	17	-5.7	9	-1.0
Liraglutide 1.8 mg	59	-7.1	15	-5.2	16	-2.2
Liraglutide 2.4 mg	65	-7.7	8	-4.6	19	-3.7
Liraglutide 3.0 mg	72	-8.4	10	-5.9	10	-3.4
Orlistat	67	-5.7	12	-0.3	16	-1.9
Placebo	67	-3.6	12	-2.6	19	-1.2

Source: FDA statistical reviewer
* Based on last available observation

A relationship was also observed between the timing of the last on-treatment assessment and the change in the primary endpoint for Trial 1839 (Figure 1) and Trial 1922 (Figure 5 in the Appendix). In particular:

- Subjects that had a 56 week on-treatment assessment (thick lines) consistently had a more favorable mean response profile over the study duration than the subjects that did not have a week 56 assessment. This observation was consistent across treatment groups.
- There was a positive relationship between the timing of the last on-treatment assessment and weight loss, with the average reduction being more favorable for subjects that had their assessment later in the trial compared to earlier.
- The distribution of the timing of the last available on-treatment was not the same across treatment arms.
- The plots do not describe what the average response at week 56 would have been for those that did not have an on-treatment assessment at week 56. For subjects that prematurely discontinued and returned for a week 56 assessment, the LAO-OT was found not to adequately characterize the week 56 response.

Figure 1. Mean profile of fasting bodyweight change (%) by last available on-treatment assessment (FAS, Trial 1839)

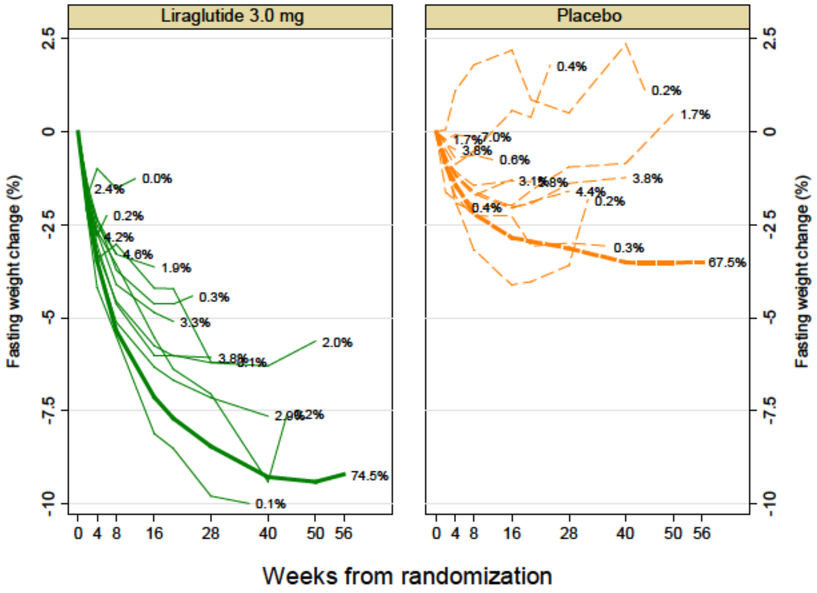

Source: FDA statistical reviewer

Missing Data in Trials 1839, 1922, and 1923: A sizable proportion of subjects did not have a have a 56 week weight assessment, with missing data occurring more frequently in the placebo group than in the liraglutide 3.0 mg group (Table 4). Across trials the proportion of missing data ranged from 17% to 20% for liraglutide 3.0 mg and from 19% to 26% for placebo. Importantly, these frequencies do not reflect the extent of missingness or treatment adherence as it relates to the primary analysis which was based on LAO-OT; the proportion of randomized subjects that did not have an on-treatment assessment at the week 56 visit ranged from 25% to 27% for liraglutide 3.0 mg and was more favorable than the 31% to 45% for placebo.

Included in the counts of subjects with a week 56 assessment are subjects that prematurely discontinued the study but returned for an assessment 56 weeks after randomization ("retrieved dropout"). The majority of subjects that prematurely discontinued did not return for the 56 week assessment, with approximately 30% of subjects doing so. In the sponsor's report on missing data they appropriately question whether subjects that did return are representative of those that did not return. It is also notable that study site also appears to impact the likelihood of returning for a follow-up assessment; sites that had a greater frequency of study discontinuations were less likely to have a follow-up assessment (Figure 2). A noteworthy example is the site that had none of the 23 subjects that discontinued returned for the 56 week assessment. How this additionally impacts the representativeness of subjects that did not return for a follow-up assessment is unclear, but it raises concern that site investigators did not uniformly adhere to the study protocol.

Table 4. Summary of missing data at week 56 (Trials 1839, 1922 and 1923)

	1839		1922			1923	
	**Lira 3.0 mg N=2487**	**Placebo N=1244**	**Lira 3.0 mg N=423**	**Lira 1.8 mg N=211**	**Placebo N=212**	**Lira 3.0 mg N=212**	**Placebo N=210**
Missing	492 (20%)	318 (26%)	67 (16%)	39 (18%)	56 (26%)	35 (17%)	39 (19%)
Available	1995 (80%)	926 (74%)	356 (84%)	172 (82%)	156 (74%)	177 (83%)	171 (81%)
On-treatment	1811 (73%)	818 (66%)	317 (75%)	158 (75%)	116 (55%)	156 (74%)	144 (69%)
Retrieve dropout	180 (7%)	103 (8%)	36 (9%)	11 (5%)	23 (11%)	21 (10%)	25 (12%)
Other‡	4 (0%)	5 (0%)	3 (1%)	3 (1%)	17 (8%)	0 (0%)	2 (1%)

Source: FDA statistical reviewer

‡ A subject that had a fasting weight measurement within the visit window for the primary landmark visit (56 weeks ± 3 days) but was neither retrieve dropout or on-treatment.

Figure 2. Relationship between having a retrieve dropout assessment and the number of discontinuations in a study site (Trial 1839)

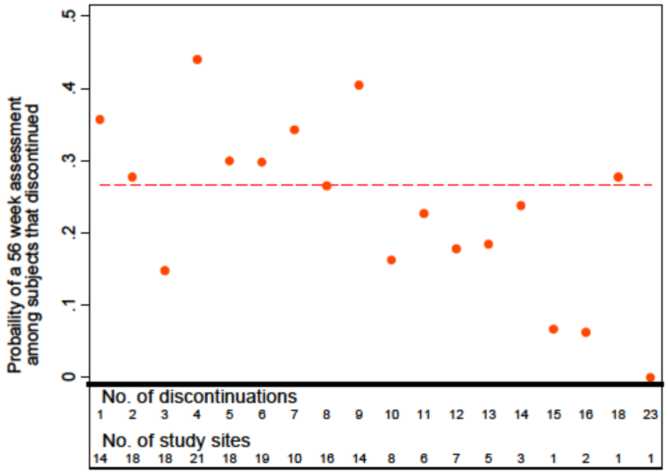

Source: FDA statistical reviewer

<u>Comparison of LAO-OT with primary endpoint</u>: This section presents findings from an empirical comparison of responses at LAO-OT and week 56 for subjects that discontinued but returned for a week 56 assessment. Notable differences between liraglutide and placebo were observed (Table 5), which include:

- For liraglutide the LAO-OT over-estimates the weight reduction at week 56, with the CI excluding the value of no difference. The proportion of subjects that maintained the weight reduction at LAO-OT was low for the 3.0 mg dose, with only 29%, 30%, and 8% doing so in Trials 1839, 1922, and 1923, respectively.
- For placebo the LAO-OT consistently under-estimated the weight reduction at week 56 although the CIs all included the value of no difference.
- The responses at week 56 had greater variability than the responses at the LAO-OT. This finding was consistent across trials and treatment groups.

These finding provide empirical confirmation that the primary analysis cannot be used to describe the ITT effect. This also extends to the analysis of categorical (responder) endpoints, for reason described next.

Table 5. Comparison of fasting weight change (%) at LAO-OT and week 56 for subjects that withdrew and returned for a week 56 follow-up assessment

Treatment Group	N	LAO-OT Mean (SE)	Week 56 (Actual) Mean (SE)	Mean Difference; LAO-OT – Week 56 (95% CI)
Trial 1839				
Liraglutide 3.0 mg	171	-4.9% (0.4)	-3.0% (0.6)	-1.8% (-2.7, -1.0)
Placebo	100	-0.4% (0.4)	-1.3% (0.7)	0.9% (-0.4, 2.1)
Trial 1922				
Liraglutide 3.0 mg	33	-4.4% (0.7)	-2.5% (0.8)	-1.8% (-3.2, -0.5)
Liraglutide 1.8 mg	8	-4.3% (1.3)	-2.4% (1.8)	-1.9% (-5.1, 1.3)
Placebo	23	-1.4% (0.4)	-1.7% (0.7)	0.3% (-1.5, 2.0)
Trial 1923				
Liraglutide 3.0 mg	12	-6.4% (1.0)	-1.1% (1.9)	-5.3% (-7.8, -2.8)
Placebo	18	-0.5% (1.0)	-1.1% (2.0)	0.5% (-2.8, 3.8)

Source: FDA statistical reviewer

Differences were observed in the frequency of responders based on LAO-OT and week 56. In Trial 1839 the proportion of 5% responders for placebo using LAO-OT under-estimated the response rate at week 56 (9% vs. 22%); for liraglutide the proportion of responses were fairly similar (LAO-OT: 34%; week 56: 32%). In Trial 1923, the proportion subjects that were able to maintain their baseline weight (i.e., the weight after a 5% reduction during the LCD run-in) was over-estimated at week 56 using LAO-OT for liraglutide (LAO-OT: 11/12; week 56: 7/12) and under-estimated using LAO-OT for placebo (LAO-OT: 7/18; week 56: 11/18).

3.3 Statistical Methods

<u>Analysis Populations</u>: Two of the sponsor's analysis populations were the full analysis set (FAS) and the completers. The FAS was the primary analysis population, and included all randomized subjects exposed to at least one dose of the trial product and with at least post-baseline assessment of body weight in Trials 1807 and 1923, or of any efficacy endpoint in Trials 1839 and 1922. The FAS in Trial 3970 was defined as all randomized subjects. This population is consistent with the modified ITT population defined in the Draft FDA Guidance (Box 2). The completer population included subjects in the FAS with a valid end of trial efficacy assessment.

The FDA analyses are performed on the ITT population, defined as randomized subjects with a baseline assessment.

All analyses use the randomized treatment.

<u>Statistical methods for the primary analysis of the primary efficacy endpoints</u>: Consistent with the Draft FDA Guidance the primary analysis was performed on the FAS using LAO-OT. In Trial 1922 the analysis was performed using last available pre-rescue observation on treatment. Continuous primary endpoints were analyzed using an analysis of covariance (ANCOVA) model that included treatment, country, sex, baseline response, and randomization stratum as independent variables. Categorical endpoints were analyzed using a logistic regression model using the same independent variables.

Note that in Trial 1922 the decision to limit the analysis to pre-rescue observation has the potential to inflate the treatment effect since subjects randomized to placebo were more likely to require rescue medication overall and earlier on average in the trial.

Sample size: The Phase 3 trials were individually powered to test the individual study endpoints with at least 85% power. The trials, in particular Trial 1839, were over-sized for the efficacy endpoints to comply with safety considerations outlined in the Draft FDA Guidance. The Guidance recommends approximately 3,000 subjects are randomized to active doses and no fewer than 1,500 subjects are randomized to placebo.

Approach to multiplicity: The Phase 3 trials (1839, 1922, 1923, 3970) individually preserved the study-wise type-I error at 5% by hierarchically testing the study endpoints according to their order in Table 2. Under this approach the statistical testing for an endpoint is performed only if the statistical test for the preceding endpoint in the hierarchy is statistically significant at the two-sided 5% level. For Trial 1922 that investigated two liraglutide doses, the hierarchy ordered the hypotheses for the 3.0 mg dose first followed by hypotheses for the 1.8 mg dose.

Approximately 15 to 20 secondary endpoints were prespecified for investigation in each of the Trials. None of the secondary endpoints, including those related to body composition in Trial 3970, were incorporated into the hierarchical testing sequence to preserve the study-wise type-I error.

For Trial 1807 the pairwise comparisons at week 20 between the separate liraglutide doses to placebo and orlistat were done using Dunnett's method for simultaneous confidence intervals. The nominal study-wise error was not preserved at the 5% level as a separate 5% alpha was used for the placebo comparison and the orlistat comparison.

Sensitivity analyses for the primary efficacy endpoints: In my opinion, the sponsor's sensitivity analyses used to assess the potential impact of missing data are inadequate. None of their analyses attempted to estimate the ITT effect at week 56 under a reasonable set of assumptions. Our recommended/preferred approach represent the missing week 56 response for subjects that prematurely discontinued using information from the subjects that also prematurely discontinued but returned for their week 56 assessment. This approach can be implemented only for Trials 1839, 1922 and 1923 because they retrieved dropouts. Additionally, I do not concur with the sponsor's definition/notion of missing data. Our notion is that all study subjects (if alive) have a weight at week 56, with their missing status being defined by whether or not the endpoint was assessed. Thus, the retrieve dropouts have a valid endpoint even though they were no longer receiving study therapy. In the sponsor's investigation of missing data the majority of their analyses did not use a subject's actual off-treatment week 56 measurements. This approach has significant implications on the interpretation of treatment effect at week 56, as detailed for the sponsor's MMRM and imputation analysis below.

***Continuous endpoints (Sponsor's)*:** Below is a description of the sponsor's sensitivity analyses that are presented in this document. With the exception of the MMRM analyses the endpoint was analyzed using an ANCOVA model using the covariates in the primary analysis.

1. *Completers* –Subset analysis that includes subjects that did not have their endpoint imputed in the primary analysis.
2. *Last available observation (LAO)* – Used fasting or nonfasting weight measurements, off-drug measurements, post-rescue and the follow-up weight measurements after 56 weeks after randomization for early withdrawal (retrieve dropout). The analysis for Trial 1923 excluded post-rescue measurements.
3. *Baseline observation carried forward (BOCF)* – Baseline observations were carried forward for subjects without a valid post-baseline assessment. This analysis was applied to all randomized subjects. This analysis was not performed in Trial 1923.
4. *MMRM* –a longitudinal analysis of on-treatment fasting weights that set off-treatment measurements to missing. A contrast and 95% CI was constructed for the difference in percent weight change for liraglutide compared to placebo at week 56.
5. *Multiple imputation (MI)* – Off-treatment responses in both treatment groups were imputed assuming the distribution of their pre- and post- withdrawal values is the same as the distribution of placebo completers. Off-treatment follow-up measurements were not included in either the imputation or the analysis.

Comments on the limitation of the sponsor's MMRM and MI analysis:
MMRM—The MMRM model assumes missing data are missing at random. Under this assumption the statistical behavior of the missing data (given the observed responses and model covariates) is assumed to be same as the observed data. Because the model uses only on-treatment observations, the model estimates the treatment effect at week 56 assuming all subjects in the FAS could adhere to randomized therapy, contrary to the fact that a sizable number could not. This analysis therefore attempts to estimate a treatment effect under conditions that were not observed in the clinical trials, nor could occur in clinical practice. Therefore, it is my opinion that the findings from this sensitivity analysis lack clinical relevance due to the underlying implausibility of achieving perfect treatment adherence.

Multiple imputation—The analysis anchors the imputed week 56 responses based on the placebo completers. Whether this is appropriate is debatable and was not justified by the sponsor. An assumption of their imputation model is, for a liraglutide treated subject, the on-treatment experiences are attributable to placebo and not the treatment received. Due to the sponsor's approach to missing data the implication of this assumption can be empirically evaluated. This was done for Trial 1839 by comparing the average imputed value with their actual value for the retrieve dropouts (Figure 3). It is evident that for liraglutide treated subjects the imputation model had them having greater average loss at week 56 than they actually did. The average decrease at week 56 from baseline was 6.1% based on the imputation, which was double the 3.0% average decrease that was actually observed and surprisingly greater than the 4.9% average decrease at the LAO-OT. For placebo the differences between imputed and observed values were not dramatic. As a consequence of these findings, it is likely that this analysis will over-state the ITT effect at week 56.

Figure 3. Kernel density plot (smoothed histogram) comparing the actual week 56 fasting weight change (%) with the average imputed value from the sponsor' MI analysis for subjects that withdrew and returned for a week 56 follow-up assessment (Trial 1839)

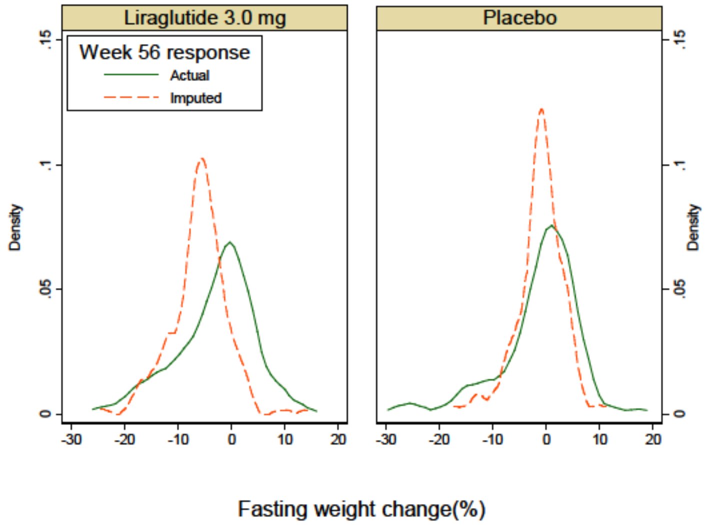

Source: FDA statistical reviewer

Categorical endpoints: Below is a description of the sponsor's sensitivity analyses that are presented in this document. Instead of comparing event probabilities using the odds ratio metric from a logistic regression model as done by the sponsor, this review will present the risk difference due to the ease of interpretation. Unadjusted estimates will be provided along with asymptotic 95% confidence interval (CI).

1. *Completers* – See description above.

2. *Off-treatment as failures* – Subjects in the FAS without a valid week 56 assessment were classified as non-responders. This analysis is consistent with a sensitivity analysis described in the Draft FDA Guidance.

Sensitivity analyses for the primary efficacy endpoints done by FDA: Two sensitivity analyses were performed by FDA to attempt to estimate the ITT effect. This was not done in Trials 1807 and 3970 since subjects that prematurely discontinued were not asked to return for an assessment at the landmark visit. How subjects were handled was not uniform across trials due to the varying number of subjects that returned for a follow-up assessment after discontinuation. Additional details of the approaches are provided in the Appendix.

Multiple imputation using retrieve dropout (MI-RD) – Our preferred approach imputes missing week 56 responses based on subjects that discontinued and had a week 56 fasting measurement. The imputation was done within groups defined by randomized treatment and the timing (month) of their last on-treatment measurement. Values were imputed using measurements from baseline and LAO-OT, when possible. This approach was not done for Trial 1923 and the liraglutide 1.8

mg arm in Trial 1922 due to the small number of retrieve dropouts; our preferred approach for Trial 1922 and comparison involving liraglutide 1.8 mg is described below.

For the continuous endpoints a total of 100 imputed datasets were created, and results were combined using Rubin's rule (Rubin, D., *Multiple Imputation for Nonresponse in Surveys*, New York: Wiley & Sons (1987)). For the categorical endpoints response status was determined from the imputed continuous response. A total of 1000 imputed data sets were created. The imputed data were analyzed using a Beta-Binomial model with a uniform prior. For each imputed dataset a sample for each group was drawn from their respective posterior distribution, which thus incorporated imputation variability. Difference in probabilities was summarized using 50th, 2.5^{th} and 97.5^{th} percentiles of the distribution.

Retrieve dropout weighted analysis (RD-Weighted) – In this analysis subjects were assigned differential weights, which up-weighted the contribution of subjects that prematurely discontinued and returned for a week 56 measurement while those missing a week 56 measurement were assigned zero weight (and did not contribute to the analysis). A subject with an on-treatment or other week 56 measurement was assigned a weight of one. The degree to which a subject was up-weighted depended on their treatment group and the timing of their LAO-OT.

For the continuous endpoints the data were analyzed using a weighted ANCOVA model. For the categorical endpoints the weighted sample was analyzed using a Beta-Binomial model with a uniform prior. A total of 100,000 samples were taken for each treatment group, and the difference in probabilities was summarized using 50th, 2.5^{th} and 97.5^{th} percentiles of the distribution.

3.4 Results

3.4.1 Trial 1807

Results from the analysis of primary endpoints at week 20 are shown below (Table 6); results for week 52 analysis are displayed in Table 14 in the Appendix. For both endpoints at week 20 only the 2.4 mg and 3.0 mg liraglutide doses had changes that were statistically significantly different than both placebo and orlistat, with the change for the 3.0 mg dose being more favorable. For the week 52 comparison the results should be interpreted extremely cautiously due to the likely bias resulting from a sizable number of subjects not consenting to the 84 week extension period. It is unclear what impact these subjects would have had if they continued in the study since they tended to have less favorable responses (Table 3).

Table 6. Analysis results for primary endpoints at week 20 in Trial 1807

Endpoint	Treatment Group	N	Adj. mean change from baseline / 5% response n (%)	Difference in means* / Risk difference Lira-Placebo (95% CI)	Difference in means* / Risk difference Orlistat-Placebo (95% CI)
Fasting weight change (kg)	Lira 3.0 mg	92	-7.2 kg	-4.4 kg (-5.9, -2.9)	-3.0 kg (-4.5, -1.4)
	Lira 2.4 mg	92	-6.3 kg	-3.5 kg (-5.0, -2.0)	-2.1 kg (-3.7, -0.6)
	Lira 1.8 mg	90	-5.5 kg	-2.8 kg (-4.3, -1.3)	-1.4 kg (-3.0, 0.2)
	Lira 1.2 mg	94	-4.8 kg	-2.1 kg (-3.6, -0.6)	-0.7 kg (-2.2, 0.9)
	Orlistat	95	-4.1 kg		
	Placebo	98	-2.8 kg		
5% responders	Lira 3.0 mg	92	70 (76%)	46.5% (33.9, 59.1)	31.9% (18.6, 45.1)
	Lira 2.4 mg	92	56 (61%)	31.3% (17.8, 44.7)	16.7% (2.5, 30.8)
	Lira 1.8 mg	90	18 (53%)	23.7% (10.0, 37.4)	9.1% (-5.2, 23.5)
	Lira 1.2 mg	94	49 (52%)	22.5% (9.0, 36.1)	7.9% (-6.3, 22.1)
	Orlistat	95	42 (44%)		
	Placebo	98	29 (30%)		

Source: FDA statistical reviewer
* Results for fasting weight are adjusted and for the 5% responder endpoint is unadjusted.

3.4.2 Trials 1839, 1922, and 1923

In each of the Phase 3 weight management trials all of the efficacy endpoints evaluated under the hierarchical testing sequence were statistically significant. To allow for a more fluid discussion of study findings the results will not be presented according to the pre-specified testing sequence. Furthermore, we caution contrasting results across trials since the trials differed in important ways with respect to study design and study population.

Change in body weight: Results from the pre-specified primary analysis of the primary efficacy endpoint is shown in Table 7. In each of the Trials liraglutide 3.0 mg treated subjects had a statistically significant greater reduction in body weight change from baseline compared to placebo. For Trials 1839 and 1922 the confidence interval did not rule out the difference in average reduction for liraglutide compared to placebo of 5%.

In Trial 1922 the liraglutide 1.8 mg treated subjects had a statistically significant greater weight reduction compared to placebo, although the difference was not as large as the reduction observed for the 3.0 mg dose.

In our preferred analysis (MI-RD for Trials 1839 and 1922, and RD-Weighted for Trial 1923) the estimate of the ITT effect remained statistically significantly better than placebo (Table 8) but the magnitude of the estimated treatment effect was attenuated relative to the primary prespecified analysis. For Trial 1839 the estimated effect was 11% smaller and 15% smaller for Trials 1922 and 1923. These findings were reasonably aligned with the second FDA sensitivity analysis that attempted to estimate the ITT effect albeit with smaller. Results from the sponsor's sensitivity analyses were found to be aligned with the findings from the primary pre-specified analysis.

Table 7. Primary analysis results for change in fasting body weight (%) in Trials 1839, 1922, and 1923

Trial	Treatment Group	N	Adj. mean change from baseline	Diff. in adj. means Lira-Placebo (95% CI)
1839	Liraglutide 3.0 mg	2432	-8.0%	-5.4% (-5.8, -4.95)
	Placebo	1220	-2.6%	
1922	Liraglutide 3.0 mg	411	-5.9%	-4.0% (-4.8, -3.1)
	Liraglutide 1.8 mg	202	-4.6%	-2.6% (-3.6, -1.6)
	Placebo	210	-2.0%	
1923	Liraglutide 3.0 mg	194	-6.1%	-6.1% (-7.5, -4.6)
	Placebo	188	-0.1%	

Source: FDA statistical reviewer

Table 8. Sensitivity analysis results for change in body weight (%) in Trials 1839, 1922, and 1923

	1839	1922		1923
**Sensitivity Analysis**	**Lira 3.0 mg - Pla. (95% CI)**	**Lira 3.0 mg - Pla. (95% CI)**	**Lira 1.8 mg - Pla. (95% CI)**	**Lira 3.0 mg - Pla. (95% CI)**
**Sponsor's**				
Completers	-5.7% (-6.3, -5.1)	-4.1% (-5.3, -2.9)	-2.7% (-4.0, -1.3)	-
LAO (FAS)	-5.2% (-5.6, -4.7)	-4.0% (-4.8, -3.1)	-2.7% (-3.7, -1.7)	-
BOCF (ITT)	-5.3% (-5.7, -4.8)	-3.8% (-4.7, -3.0)	-2.4% (-3.4, -1.4)	-5.4% (-6.8, -3.9)
MMRM (FAS)	-5.8% (-6.3, -5.3)	-4.4% (-5.5, -3.3)	-2.9% (-4.2, -1.7)	-6.1% (-7.7, -4.6)
MI (FAS)	-5.5% (-6.0, -5.0)	-4.0% (-5.1, -2.9)	-2.7% (-4.0, -1.4)	-
**FDA**				
MI-RD (ITT)	-4.8% (-5.3, -4.3)	-3.4% (-4.5, -2.3)	-	-
RD-Weighted (ITT)	-4.6% (-5.4, -3.9)	-3.8% (-4.7, -2.9)	-2.5% (-3.5, -1.5)	-5.3% (-6.8, -3.8)

Source: FDA statistical reviewer

Responder endpoints: Results from the pre-specified primary analysis of the responder endpoints is shown in Table 7. In each trial for each of the two responder endpoints, the liraglutide 3.0 mg treated subjects had a statistically significant excess number of subjects respond compared to placebo. For Trials 1839 and 1922 the estimated proportion of liraglutide 3.0 mg treated subjects having a 5% response were notably greater than 35% and more than double the proportion in placebo.

In Trial 1922 the liraglutide 1.8 mg treated subjects also had a statistically significant excess number of subjects responders compared to placebo. The estimated proportion of liraglutide 1.8 mg treated subjects having a 5% response was similar to 35% (36%) and more than double the proportion in placebo.

In our preferred analysis the estimate of the ITT effect remained statistically significantly better than placebo (Table 10) but, similar to the findings from the continuous endpoint, the magnitude of the estimated treatment effect was attenuated relative to the primary prespecified analysis.

For Trials 1839 and 1922 this attenuation can be attributed the statistical model predicting a greater number placebo treated subjects having a 5% response compared to LAO-OT (Trial 1839: 34% vs. 27%; Trial 1922: 20% vs. 14%). For these two trials the estimated proportion of liraglutide 3.0 mg treated subjects having a 5% response remained above 35% and approximately double the proportion in placebo.

Table 9. Primary analysis results for responder endpoints in Trials 1839, 1922, and 1923

Trial	Responder Endpoint	Treatment Group	N	n (%)	Difference* Lira-Placebo (95% CI)	Odds Ratio* Lira/Placebo (95% CI)
1839	5%	Lira 3.0 mg	2432	1536 (63%)	36.0% (32.9, 39.2)	4.8 (4.1, 5.6)
		Placebo	1220	331 (27%)		
	10%	Lira 3.0 mg	2432	805 (33%)	22.5% (20.0, 25.1)	4.3 (3.5, 5.3)
		Placebo	1220	129 (11%)		
1922	5%	Lira 3.0 mg	411	205 (50%)	36.1% (29.4, 42.8)	6.8 (4.3, 10.7)
		Lira 1.8 mg	202	72 (36%)	21.8% (13.7, 29.9)	3.7 (2.2, 6.1)
		Placebo	210	29 (14%)		
	10%	Lira 3.0 mg	411	96 (23%)	19.1% (14.1, 24.0)	7.1 (3.5, 14.5)
		Lira 1.8 mg	202	29 (14%)	10.1% (4.5, 15.6)	3.8 (1.8, 8.4)
		Placebo	210	9 (4%)		
1923	Maintain	Lira 3.0 mg	194	158 (82%)	32.5% (23.5, 41.5)	4.8 (3.0, 7.7)
		Placebo	188	92 (50%)		
	5%	Lira 3.0 mg	194	98 (51%)	28.7% (19.5, 37.9)	3.9 (2.4, 6.1)
		Placebo	188	41 (22%)		

Source: FDA statistical reviewer

* Odds ratio estimates are from an adjusted analysis while the estimated risk difference is unadjusted

Table 10. Sensitivity analysis results for responder endpoints in Trials 1839, 1922, and 1923

	**1839**			**1922**			**1923**		
**Endpoint/ Sensitivity Analysis**	**Lira 3.0mg n (%)**	**Placebo n (%)**	**Difference: Lira - Placebo (95% CI)**	**Lira 3.0mg n (%)**	**Placebo n (%)**	**Difference: Lira - Placebo (95% CI)**	**Lira 3.0mg n (%)**	**Placebo n (%)**	**Difference: Lira - Placebo (95% CI)**
**5% responder**									
Completers	1317 (73%)	292 (36%)	37% (33, 39)	186 (59%)	24 (21%)	38% (29, 47)	83 (53%)	32 (22%)	31% (21, 41)
Fails (FAS)	1317 (54%)	292 (24%)	30% (27, 33)	186 (45%)	24 (11%)	34% (27, 40)	83 (43%)	32 (17%)	26% (17, 35)
MI-RD (ITT)	1542 (62%)	420 (34%)	28% (24, 32)	211 (50%)	40 (20%)	31% (22, 39)	94 (44%)	47 (23%)	22% (12, 31)
RD Weights (ITT)	1528 (62%)	381 (31%)	31% (28, 34)	215 (51%)	31 (15%)	36% (29, 42)	94 (44%)	44 (21%)	23% (14, 31)
**10% responder**									
Completers	739 (41%)	122 (15%)	26% (23, 29)	87 (27%)	9 (8%)	20% (13, 27)	-	-	-
Fails (FAS)	739 (30%)	122 (10%)	20% (18, 23)	87 (21%)	9 (4%)	17% (12, 22)	-	-	-
MI-RD (ITT)	841 (34%)	186 (15%)	19% (15, 22)	95 (23%)	14 (7%)	16% (9, 21)	-	-	-
RD Weights (ITT)	855 (34%)	174 (14%)	20% (18, 23)	98 (23%)	13 (6%)	17% (12, 22)	-	-	-
**Maintain**									
Completers	-	-	-	-	-	-	126 (81%)	69 (48%)	33% (23, 43)
Fails (FAS)	-	-	-	-	-	-	126 (65%)	69 (37%)	28% (19, 38)
MI-RD (ITT)	-	-	-	-	-	-	-	-	-
RD Weights (ITT)	-	-	-	-	-	-	152 (72%)	94 (45%)	27% (18, 36)

Source: FDA statistical reviewer

Cumulative distribution plots were constructed to allow investigating of different thresholds beyond those considered above. (Plots for Trials 1922 and 1923 are displayed in the Appendix.) Importantly, randomized subjects that were no longer on-treatment by week 56 and/or did not have an endpoint assessment were assigned the worst possible weight change. This resulted in the initial step in the curves, but removed the potential of having time-confounded curves. The expectation in such a plot is that if liraglutide was not efficacious the liraglutide curve would be similar or worse (due to potential adverse effects) than placebo over the changes from baseline that are considered meaning (e.g., > 5%). This was not what was observed, with the proportion of responders being greater in the liraglutide group.

This plot also enables one to answer the following question regarding a treatment decision: For a patient considering treatment with liraglutide for 56 weeks, how likely are they to stay on treatment for the intended duration and experience a change in fasting weight of a certain degree. Such a question could not be answered from a plot using LAO-OT.

Figure 4. Empirical distribution plot of being on-treatment and fasting weight change (%) at week 56 (all randomized, Trial 1839)

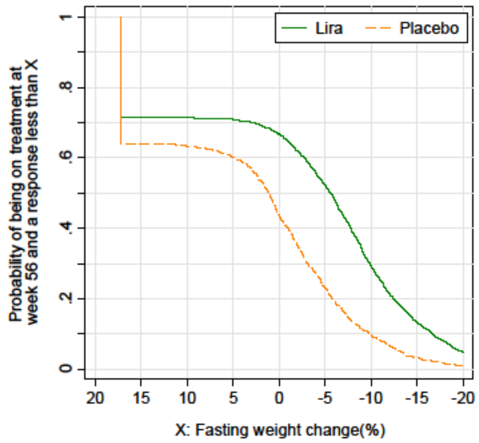

Source: FDA statistical reviewer

3.4.3 Trial 3970

Results from the analysis of the primary efficacy endpoint (AHI) and the secondary body weight endpoints are shown in Table 11. For on-treatment changes in AHI up until week 32, liraglutide treated subjects had a statistically significant greater reduction from baseline relative to placebo; the excess reduction was -6.1 events/per hour with 95% CI (-11.0, -1.2). Based on previous discussions it is unclear whether this reduction is clinically relevant.

For the weight endpoints, compared to placebo by week 32, the liraglutide treated subjects experienced an additional decrease in body weight of 4.2%, and an estimated additional 27.7 and 21.7 subjects per 100 treated that would have had weight reductions of at least 5% and 10%, respectively.

Table 11. Analysis results for change in AHI (events/hour) and secondary weight endpoints in Trial 3970

Endpoint	Treatment Group	N	Adj. mean change from baseline/ response n (%)	Diff. in means* Lira-Placebo (95% CI)
AHI	Liraglutide 3.0 mg	168	-12.2	-6.1 (-11.0, -1.2)
	Placebo	166	-6.1	
% change	Liraglutide 3.0 mg	175	-5.7%	-4.2% (-5.2, -3.1)
	Placebo	178	-1.6%	
5% responders	Liraglutide 3.0 mg	175	81 (46%)	27.7% (18.4, 37.1)
	Placebo	178	33 (19%)	
10% responders	Liraglutide 3.0 mg	175	41 (23%)	21.7% (15.2, 28.3)
	Placebo	178	3 (2%)	

Source: FDA statistical reviewer

* Results for AHI and fasting weight change (%) are adjusted and the responder endpoints are unadjusted.

4 Summary results

Based on our preferred analysis subjects treated with liraglutide were found to have statistically significant changes in body weight. Compared to placebo, the excess reduction in fasting weight from baseline to week 56 for liraglutide 3.0 mg was 4.8% (95% CI =4.3, 5.3) in Trial 1839 and 3.4% (95% CI =2.3, 4.5) in Trial 1922. When liraglutide was used after an initial 5% weight reduction from a LCD, liraglutide 3.0 mg treated subjects lost an additional 5.3% (95% CI =3.8, 6.8) compared to placebo. Although the magnitude of the estimated treatment effects from our preferred approach were attenuated relative to the pre-specified primary analysis, the changes that were observed for liraglutide 3.0 mg relative to placebo were in-line with the efficacy benchmarks outlined in the 2007 Draft FDA Guidance.

5 APPENDIX

5.1 Supportive Material

<u>Definition of obstructive apnea and hypopnea events</u> per study protocol (Section 3.2)

Apnea Rules

Score an apnea when all of the following criteria are met:

- There is a drop in the peak thermal sensor excursion by ≥90% of baseline
- The duration of the event lasts at least 10 seconds
- At least 90% of the event's duration meets the amplitude reduction criteria of apnoea

Hypopnea Rules

Score a hypopnea if all of the following criteria are met:

- The nasal pressure signal excursions (or those of the alternative hypopnea sensor) drop by ≥30% of baseline
- The duration of this drop occurs for a period lasting at least 10 seconds
- There is a ≥4% desaturation from pre-event baseline
- At least 90% of the event's duration must meet the amplitude reduction of criteria for hypopnea

<u>Details of the FDA sensitivity analyses</u>

MI-RD –The imputation was done within groups defined by randomized treatment and the timing (month) of their last on-treatment measurement. In Trial 1839 the visits were grouped by month as follows: 0 to 1, 2 to 3, 4 to 6, 7 to 9, after 10. In Trial 1922 the visits were grouped based on whether the last on-treatment measurement was on or before month 5. For subjects in the FAS the imputation model, fit within each group, included baseline and last on-treatment measurement. Imputation for randomized subjects excluded from the FAS was done as follows. These subjects were first grouped with the subjects that had their last on-treatment measurement during the first time period (Trial 1839: 0 to month 1; Trial 1922: 0 to month 5). In the first step the missing week 56 response was imputed using only their baseline measurement. Next, the distribution of imputed values was centered per subject around their baseline measurement (i.e., MI version of BOCF).

RD-Weighted – Subjects with a week 56 assessment that were not a retrieve dropout were assigned an analysis weight of one. Subjects without a week 56 assessment were assigned an analysis weight of 0. The retrieve dropouts were assigned weights that depended on the time of their last on-treatment observation and randomized treatment. Specifically, the analysis weight assigned to a subject that was a retrieve dropout in group *i* was $(A_i + B_i)/A_i$ where A_i is the number of retrieve dropouts in the group and B_i is the number of subjects in the group with the missing endpoint. For Trial 1839 and 1922 the timing used to define the groups was based on the MI-RD analysis (see above). In Trial 1923 the visits were grouped based on whether the last on-treatment measurement was on or before month 4

5.2 Additional Tables and Figures

Figure 5. Mean profile of fasting bodyweight change (%) by last available on-treatment follow-up visit (FAS, Trial 1922)

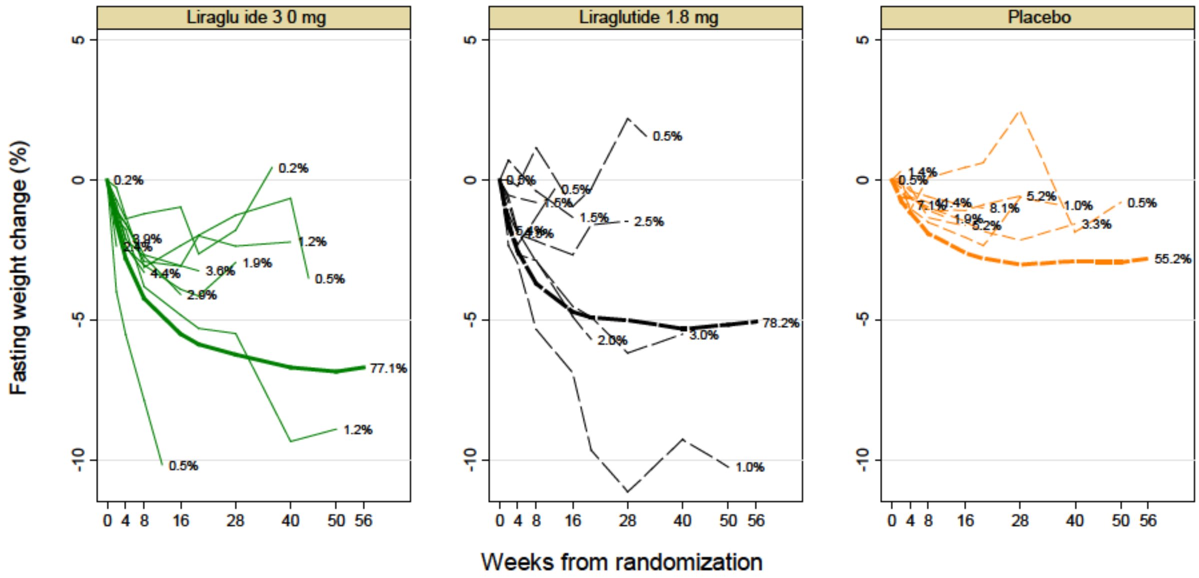

Source: FDA statistical reviewer

Figure 6. Empirical distribution plot of being on-treatment and fasting weight change (%) at week 56 (all randomized, Trial 1922)

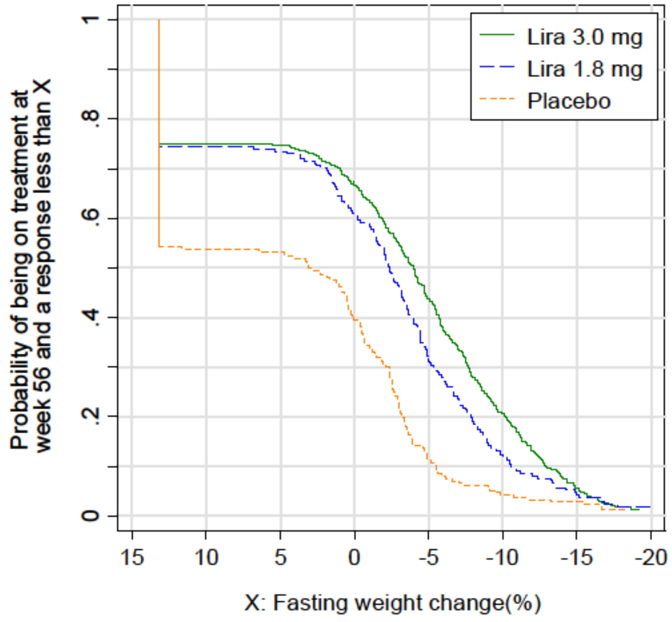

Source: FDA statistical reviewer

Figure 7. Empirical distribution plot of being on-treatment and fasting weight change (%) at week 56 (all randomized, Trial 1923)

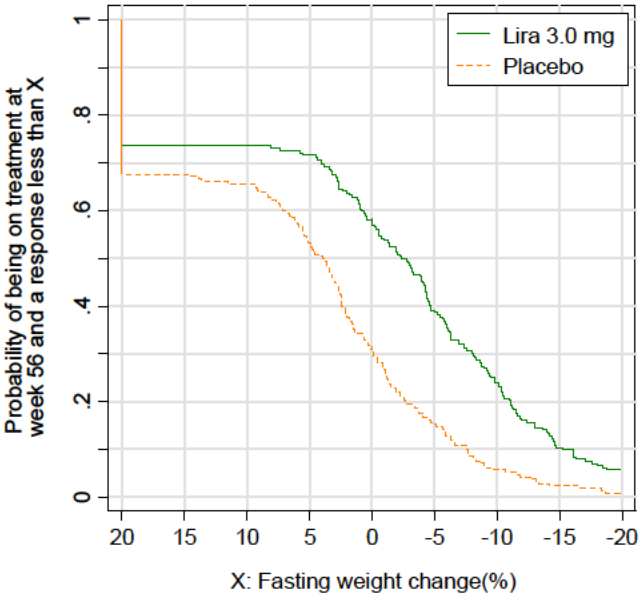

Source: FDA statistical reviewer

Table 12. Patient Disposition by trial

	1807			1839		1922			1923		3970	
	**Lira 3.0 N**	**Orlistat N**	**Placebo N**	**Lira 3.0 N**	**Placebo N**	**Lira 3.0 N**	**Lira 1.8 N**	**Placebo N**	**Lira 3.0 N**	**Placebo N**	**Lira 3.0 N**	**Placebo N**
Randomized	93	95	98	2487	1244	423	211	212	212	210	180	179
Exposed	93	95	98	2481	1242	422	210	212	212	210	176	179
Completed treatment period*	82	79	79	1789	801	324	164	140	159	146	134	142
Withdrawn*	11	16	19	698	443	99	47	72	53	64	46	37
Adverse event	5	3	3	238	45	39	18	7	18	18	20	6
Ineffective therapy	0	1	2	23	36	0	0	3	0	2	2	1
Non-compliance with protocol	2	2	3	65	38	12	8	13	8	5	8	5
Other	4	10	11	79	63	16	7	12	10	15	14	25
Withdrawal criteria	0	0	0	293	261	32	14	37	17	24	2	0
Consented to 84 Week Extension	72	67	67	-	-	-	-	-	-	-	-	-
Interim Period (Weeks 20 – 52)												
Completed	65	55	62	-	-	-	-	-	-	-	-	-
Withdrawn	7	12	5	-	-	-	-	-	-	-	-	-
Adverse event	2	0	0	-	-	-	-	-	-	-	-	-
Ineffective therapy	0	0	2	-	-	-	-	-	-	-	-	-
Non-compliance with protocol	0	1	0	-	-	-	-	-	-	-	-	-
Other	5	11	3	-	-	-	-	-	-	-	-	-
Withdrew but attended 1yr visit	-	-	-	202	111	36	12	23	22	25	-	-
Entered re-randomization	-	-	-	701	304	-	-	-	-	-	-	-
Completed re-randomization	-	-	-	685	289	-	-	-	-	-	-	-
Full analysis set	92	95	98	2437	1225	412	204	211	207	206	180	179

Source: FDA statistical reviewer

% of randomized subjects; *During 20 week main treatment period for Trial 1807

Table 13. Select instances of withdrawal criteria related to inadequate weight loss (Trial 1839)

Subject ID	Reason noted in dataset
440012	Subject is tired of daily injections without weight loss over the year of participation
446016	WITHDREW BECAUSE SUBJECT WAS NOT LOSING WEIGHT
440026	Subject did not care to commit time and effort to study since she was not losing significant weight and did not want to continue daily injections.
445001	Weight loss stopped..Patient does not want to continue giving injections for no weight loss
446001	WITHDREW CONSENT BECAUSE SUBJECT WAS NOT LOSING WEIGHT
446010	WITHDREW CONSENT BECAUSE SUBJECT WAS NOT LOSING ANY WEIGHT
446011	WITHDREW CONSENT BECAUSE SUBJECT WAS NOT LOSING ANY WEIGHT

Source: FDA statistical reviewer

Table 14. Analysis results for primary endpoints at week 52 in Trial 1807

Endpoint	Treatment Group	N	Adj. mean change from baseline / 5% response n (%)	Difference in means / Risk difference Lira-Placebo (95% CI)	Difference in means / Risk difference Orlistat-Placebo (95% CI)
Fasting weight change (kg)	Lira 3.0 mg	92	-7.8 kg	-5.8 kg (-7.9, -3.7)	-3.8 kg (-6.0, -1.6)
	Lira 2.4 mg	92	-6.1 kg	-4.1 kg (-6.2, -2.0)	-2.2 kg (-4.4, -0.0)
	Lira 1.8 mg	90	-5.4 kg	-3.4 kg (-5.5, -1.2)	-1.5 kg (-3.7, 0.7)
	Lira 1.2 mg	94	-3.8 kg	-1.8 kg (-3.9, 0.4)	0.2 kg (-2.0, 2.4)
	Orlistat	95	-3.9 kg		
	Placebo	98	-2.0 kg		
5% responders	Lira 3.0 mg	92	68 (74%)	45.3% (32.7, 58.0)	28.6% (15.2, 42.1)
	Lira 2.4 mg	92	49 (53%)	24.7% (11.1, 38.3)	8.0% (-6.3, 22.3)
	Lira 1.8 mg	90	47 (52%)	23.7% (10.0, 37.3)	7.0% (-7.4, 21.3)
	Lira 1.2 mg	94	42 (45%)	16.1% (2.7, 29.6)	-0.6% (-14.8, 13.6)
	Orlistat	95	43 (45%)		
	Placebo	98	28 (29%)		

Source: FDA statistical reviewer
